# Human rhinovirus 16 induces an ICAM-1-PKR-ATF2 axis to modulate macrophage functions

**DOI:** 10.1128/jvi.01499-24

**Published:** 2024-09-26

**Authors:** Suzanne Faure-Dupuy, Manon Depierre, Zoé Fremont-Debaene, Floriane Herit, Florence Niedergang

**Affiliations:** 1 Université Paris Cité, Institut Cochin, INSERM, CNRS, Paris, France; St. Jude Children's Research Hospital, Memphis, Tennessee, USA

**Keywords:** macrophages, rhinovirus, interferon, ARL5b, epigenetic regulation, host-pathogen interaction, ATF2, PKR, ICAM-1

## Abstract

Human rhinovirus (HRV) infections are the leading cause of disease exacerbations in individuals with chronic pulmonary diseases, primarily due to impaired macrophage functions, resulting in defective bacterial elimination. We previously demonstrated that HRV16 impairs macrophages’ functions in an ARL5b-dependent manner. In permissive cells, ARL5b acted as an HRV16 restriction factor and was repressed. Here, we delve into the dual regulation of ARL5b by HRV16 in both cell types. We analyzed the effect of HRV16 on primary human macrophages using neutralizing antibodies, specific inhibitors, siRNA, and chromatin immune precipitation. Our study reveals that, while the virus does not replicate in macrophages, it induces interferon and pro-inflammatory responses. We identify the ICAM-1-PKR-ATF2 signaling axis as crucial for ARL5b induction in macrophages, whereas only ICAM-1 plays a role in ARL5b repression in permissive cells. Furthermore, HRV16 triggers epigenetic reprogramming in both cell types at the ARL5b promoter. In macrophages, epigenetic changes are ATF2 dependent. In conclusion, our findings highlight previously unknown signaling pathways activated by HRV16 in macrophages. Targeting these pathways could offer novel strategies to improve outcomes for individuals with respiratory conditions.

**IMPORTANCE:**

Human rhinovirus (HRV) infections are the leading cause of disease exacerbations in individuals with chronic pulmonary conditions and are frequently associated with bacterial superinfections due to defective bacterial elimination by macrophages. We previously identified ARL5b-induction by HRV16 to be responsible for the impairment of bacteria elimination. In contrast, in permissive cells, ARL5b is repressed and acts as a restriction factor for HRV16. Here, we investigated the dual regulation of ARL5b by HRV16 in these cells. Our study reveals that the ICAM-1-PKR-ATF2 signaling axis is crucial for ARL5b induction in macrophages. In permissive cells, only ICAM-1 plays a role in ARL5b repression. Moreover, HRV16 triggered epigenetic reprogramming in macrophages. ARL5b promoter was repressed in an ATF2-dependent manner. Collectively, our findings reveal previously unknown signaling pathways activated by HRV16 in macrophages. Targeting these pathways provides novel strategies to target ARL5b expression specifically in macrophages and improve outcomes for individuals with respiratory pathologies.

## INTRODUCTION

Human rhinoviruses (HRV) are the causative agents of the common cold (1). These non-enveloped viruses possess an icosahedral capsid containing a single-stranded positive-sense RNA genome (1). Over 160 serotypes, categorized into three species (HRV-A, HRV-B, and HRV-C), have been identified to date. These can be further classified into three groups based on the receptor used for cellular entry. HRV-A and HRV-B are divided into a major group, utilizing intercellular adhesion molecule-1 (ICAM-1) for entry, and a minor group, utilizing the low-density lipoprotein receptor (LDLR) (1). HRV-C serotypes enter cells through Cadherin-Related Family Member 3 (CDHR3) (2). Notably, HRV16, a commonly studied strain, belongs to the major group of the HRV-A species.

While HRV typically infects the upper respiratory tract, in patients with chronic inflammatory respiratory diseases like chronic obstructive pulmonary disease (COPD) or asthma, HRV can also infect the lower respiratory tract (3). Infections in the lower respiratory tract have been linked to disease exacerbations and bacterial superinfections (4–6). Studies, including ours, have demonstrated that bacterial superinfections are associated with impaired phagocytosis in macrophages (7–13).

Alveolar macrophages, as the first line of defence in the lungs, can phagocytose material and cell debris through specific surface receptors (14, 15). Our previous results demonstrated that HRV16 affects phagocytic uptake in an Arpin-dependent manner (9) and bacterial clearance in an ADP Ribosylation Factor-Like GTPase 5B (ARL5b)-dependent manner (13). Interestingly, in macrophages, ARL5b expression increases upon viral contact, while it decreases in HeLa OHIO cells, a model used to replicate HRV16. In HeLa OHIO cells, depletion of ARL5b increased the secretion of infectious virions, while ectopic expression of ARL5b led to a decrease in viral production, suggesting ARL5b acts as a restriction factor (13). The mechanisms governing this dual regulation of ARL5b in HeLa OHIO and human macrophages are yet to be defined.

Previous studies have reported that HRV16 triggers an immune response in human macrophages, but the extent of this response and the cellular pathways involved remain unclear (16–18). Furthermore, while HRV has been shown to replicate in epithelial cells (13, 19), its potential replication in macrophages has yet to be fully elucidated (9, 20).

Upon detecting pathogens, cells can be activated, leading to the production of cytokines and interferons (21). The protein kinase R (PKR) plays a crucial role in various signaling pathways, responding to bacterial or viral infections and cytokine detection (21–23). PKR can phosphorylate different effectors, including mitogen-activated protein kinases (MAPK) and IκB kinase (IKK) (23), leading to the activation of transcription factors such as c-Jun, ATF2, and the NF-κB pathway (23). Activated transcription factors translocate to the nucleus, influencing gene expression through interactions with the epigenetic status of gene promoters and enhancers, and subsequent DNA compaction (24, 25). Various epigenetic marks have been described. Among others, the acetylation of lysine 27 of histone 3 (H3K27Ac) is an activating mark, whereas the trimethylation of lysine 27 of histone 3 (H3K27Me3) is dependent on the methyltransferase enhancer of zeste homolog 2 (EZH2) is a repressive mark (26).

In this study, we analyzed the immune activation triggered by HRV16 in macrophages to elucidate the cellular pathways involved in ARL5b upregulation. We demonstrate that HRV16 does not replicate in macrophages but induces interferon and pro-inflammatory responses. Conversely, in HeLa OHIO cells where the virus replicates, no induction of immune responses is observed. We identify the ICAM-1-PKR-ATF2 axis as the primary regulator of HRV16-mediated ARL5b induction in macrophages. By contrast, the small HRV16-mediated ARL5b decrease in HeLa OHIO is dependent on ICAM-1/viral entry in the cells but not on PKR signaling. Finally, ARL5b is regulated at the epigenetic level in both HeLa OHIO and human macrophages. An increase in the repressive mark H3K27Me3 is observed in HeLa OHIO, while the activating mark H3K27Ac is increased on the ARL5b promoter in human macrophages.

## MATERIALS AND METHODS

### Cell culture

Whole blood of healthy donors was obtained from the Etablissement Français du Sang (INSERM agreement #18/EFS/030) ensuring that all donors gave a written informed consent and provided anonymized samples. Peripheral blood mononuclear cells (PBMCs) were isolated through a Ficoll-Paque (GE Healthcare) density gradient. Cells were further isolated by a 56% Percoll density gradient (GE Healthcare). PBMCs were plated in the presence of 4 ng/mL GM-CSF and 0.5 ng/mL M-CSF in R10 medium [RPMI 1640 (Life Technologies) supplemented with 10% FBS (Gibco), 10 mM HEPES (Gibco), 1 mM sodium pyruvate (Gibco), 100 µg/mL penicillin/streptomycin (Gibco), and 1× non-essential amino acids (Gibco)]. Medium and cytokines were replaced 4 days post plating. Seven days post plating, the medium was changed to macrophage medium [RPMI 1640 (Life Technologies) supplemented with 10% FCS (Gibco), 100 µg/mL penicillin/streptomycin (Gibco), and 2 mM L-glutamine (Gibco)]. After 10 days of differentiation, primary human monocyte-derived macrophages (hMDMs) were used as described in the figures.

HeLa OHIO cells were purchased from the European Collection of Authenticated Cell Cultures (ECACC) and were cultured in complete HeLa OHIO medium [DMEM GlutaMax with 25 mM D-glucose (Life Technologies) supplemented with 10% FCS (Gibco), 100 µg/mL penicillin/streptomycin, and 2 mM L-glutamine (Gibco)]. Cells were passaged three times a week.

### Human rhinovirus production

Human rhinovirus 16 (HRV16) (VR-283, strain 11757, lot 62342987) was purchased from the ATCC. To produce viral stocks, HeLa OHIO cells were grown to 80% confluence in six well plates and infected in 300 µL of virus medium (DMEM GlutaMax containing 25 mM D-glucose supplemented with 10% FCS and 2 mM L-glutamine) in the presence of 0.25 × 10^7^ TCID_50_/mL HRV16 or control medium (Mock infected or MI). Infection was performed at room temperature (RT) for 1 h with agitation. Then, the medium was completed to a final volume of 2 mL per well. Once the cytopathic effect reached 90% of the cells in the HRV16 condition, cultures were frozen and thawed three times. Supernatants were collected, centrifuged for 15 min at 3,900 rpm, and filtered at 0.22 µm. 1 mL stocks were generated and kept at −80°C.

### Quantification of the tissue culture infectious dose 50 (TCID_50_) of HRV16

Fifteen thousand HeLa OHIO cells were plated per well in a 96 well plate. After 48 h, cells were infected with HRV16 or MI. HRV16 and MI were diluted 10-fold from undiluted to 10^−9^ in virus medium, and 100 µL of each dilution was added to the cells in six replicate wells for HRV16 and two replicate wells for MI. One hundred microliters of virus medium was added to eight replicate wells as a control. Cells were cultured at 37°C until a cytopathic effect was observed in 50% of the wells (72 h on average). TCID_50_ was calculated using the Spearman-Karber formula.

### HRV16 infection

Macrophages or HeLa OHIO were washed once in virus medium. HRV16 or MI stocks were added to the cells to achieve 1 × 10^7^ TCID_50_/mL, which is equivalent to a MOI of 28 for hMDMs and a MOI of 14 for HeLa OHIO. MOI was calculated based on the number of monocytes plated for differentiation into hMDMs and the number of HeLa OHIO counted for plating the day before the infection. Alternatively, for the indicated experiments, HRV16 and MI were diluted for the infection as follows: Dose 1 = 1 × 10^7^ TCID_50_/mL, Dose 2 = 5 × 10^6^ TCID_50_/mL (MOI of 14 for hMDMs, MOI of 7 for HeLa OHIO), Dose 3 = 2.5 × 10^6^ TCID_50_/mL (MOI of 7 for hMDMs, MOI of 3.5 for HeLa OHIO), Dose 4 = 12.5 × 10^6^ TCID_50_/mL (MOI of 3.5 for hMDMs, MOI of 1.75 for HeLa OHIO). Infection was performed at RT for 1 h with agitation. Cells were then washed with virus medium and further cultured in macrophage medium or HeLa OHIO medium, respectively, for the indicated amount of time.

### Cytokines, inhibitors, and antibodies treatments

Differentiated hMDMs or HeLa OHIO were infected or not with HRV16 to achieve the indicated TCID_50_. After contact for 1 h at RT, cells were treated for 24 h with four different doses of interferon beta (IFNβ; Peprotech, 300–02B.C.BC) as follows: Dose 1 = 1 µg/mL, Dose 2 = 100 ng/mL, Dose 3 = 10 ng/mL, Dose 4 = 1 ng/mL.

Differentiated hMDMs were pre-treated with 50 µM C16 (inhibitor of PKR; Merck 527450-5MG) or with an equivalent amount of DMSO as control for 1 h. Then, cells were washed with macrophage medium and infected or not as indicated.

Differentiated hMDMs or HeLa OHIO were incubated with an antibody against human ICAM-1 (Biotechne, BBA3) or a control IgG antibody (Abcam, ab170190) at a final concentration of 10 µg/mL for 1 h before and during the infection with HRV. Then, cells were washed with macrophage medium. Alternatively, after the infection, the medium containing the virus was removed, then the cells were washed on ice with acidic medium (RPMI 1640 (Life Technologies) supplemented with 50 mM of NaAc, pH 2) for 2 min and further washed with PBS. The two washes were repeated once before cell lysis.

### siRNA transfection

Differentiated hMDMs were transfected with control siRNA directed against Luciferase (CGUACGCGGAAUACUUCGA), or siRNAs against ATF2 (1: GCUUCAGAAGAUGACAUUA; 2: GGAAGUACCAUUGGCACAA). Shortly, for a well of 24-wells plate, 0.1 mL of Opti-MEM (Gibco) was mixed with 100 nM of the indicated siRNA (or 50 nM of each siRNA in case of combination of two siRNAs) and with 0.8 µL of Lipofectamine RNAiMax (Invitrogen) in this order. The mix was inverted five times and incubated for 20 min at RT. In the meantime, the culture medium of the differentiated hMDMs was replaced with fresh medium. Then, siRNA mix was added drop by drop to the cells. Plate was rocked softly to mix and incubated at 37°C for 72 h.

### RNA extraction

After the indicated infection time and/or treatment, macrophages or HeLa OHIO were washed once with PBS and lysed in 350 µL of LBP (Macherey-Nagel, 740984.250). Extraction was performed following the manufacturer’s instructions.

### Reverse transcription

RNAs were quantified using a NanoDrop 2000 Spectrophotometer. mRNA was reverse transcribed using a high-capacity cDNA reverse transcription kit (ThermoFisher Scientist, 4368813). The reaction was performed using 500 ng of mRNA in 10 µL, 2 µL of 10× RT Buffer, 0.8 µL of 25× dNTP Mic (100 mM), 2 µL of 10× Random Primers, 1 µL of MultiScribe Reverse Transcriptase, and 4.2 µL of nuclease-free H_2_O. Samples were put in a thermocycler and the following steps were applied: 10 min at 25°C, 120 min at 37°C, 5 min at 85°C, and 4°C on hold.

### LDH test

During the experiments, supernatants were collected and kept at −20°C. To evaluate cytotoxicity, supernatants were analyzed for LDH release using the CyQUANT LDH Cytotoxicity Assay Kit, following the manufacturer’s instructions. Positive controls were obtained using 1× Lysis Buffer provided in the kit.

### Chromatin immuno-precipitation

First, DNA and proteins were crosslinked as follows: cells were washed once with PBS, then incubated with 1% PFA with agitation for 10 min at RT, and washed once with PBS 1×. To stop the cross-linking, cells were incubated with agitation 5 min with 0.125 M glycine diluted in PBS before being washed once with PBS 1×. Cells were scraped in 1 mL PBS 1× and collected and centrifuged at 3,500 rpm for 10 min at 4°C. Pellets were washed once with PBS 1× + 1 mM PMSF + 1× protease inhibitors cocktail (PIC; Roche Diagnostic, 11836170001). Cells were centrifuged as before and pellets were kept at −80°C or lysed in lysis buffer (50 mM Tris HCL pH8, 10 mM EDTA, 1% SDS, 1× PIC, 1 mM PMSF). Cells were sonicated to obtain fragments between 300 and 500 bp and centrifuged at 13,000 rpm for 10 min at 4°C. Supernatants were pre-cleared by incubating them with Protein A Sepharose beads for 2 h at 4°C with rotation. Protein A Sepharose beads were discarded by a 1 min centrifugation at 1,200 rpm and at 4°C. Antibodies were added to the samples, and the samples were rotated at 4°C O/N. The following antibodies were used: anti-Tri-Methyl-Histone H3 (Lys27) (diluted 1:50; Cell Signaling Technologies, 9733S), anti-H3K27Ac (1 µg/sample; Diagenode C15410196), anti-EZH2 (diluted 1:100; Cell Signaling Technologies, 5246S), control Rabbit IgG (1:100; Cell Signaling Technologies, 2729S). The next day, the samples were incubated with magnetic beads coated with protein A for 2 h at 4°C with rotation. Subsequently, the magnetic beads were washed four times with RIPA buffer (10 mM Tris HCl pH 7.5, 140 mM NaCl, 1 mM EDTA, 0.5 mM EGTA, 1% Triton, 0.1% SDS, 0.1% Na-Deoxycholate, 1× PIC, 1 mM PMSF), one time with LiCl buffer (0.25 M LiCl, 0.5% NP40, 0.5% Na-deoxycholate, 10 mM Tris HCl pH 8, 1 mM EDTa, 1× PIC, 1 mM PMSF), and one time with TE buffer (10 mM Tris HCl pH 8, 10 mM EDTA). The cross-linking was reversed in Elution buffer (20 mM Tris HCl pH 7.5, 5 mM EDTA, 50 mM NaCl, 1% SDS, 50 µg/mL Proteinase K) at 68°C for 2 h with 1,300 rpm agitation. DNA was extracted in Phenol:Chloroform:Isoamyl Alcohol (25:24:1) and resuspended in DNase free water.

### qPCR

qPCR was performed using SensiFAST SYBR No-ROX Kit (biotechnofix, BIO-98050) and specific primers detecting the indicated genes. TBP was used as a house-keeping gene. All primers are listed in Table S1.

### Western blots

HeLa OHIO were lysed in NP-40 lysis buffer (20 mM Tris HCl, pH 7.5, 150 mM NaCl, 0.5% NP-40, 50 mM NaF, and 1 mM sodium orthovanadate) supplemented with a protease inhibitor cocktail (cOmplete, Roche) for 15 min on ice. Lysates were centrifuged at 13,000 rpm for 10 min at 4°C. The supernatants were collected and stored at −20°C until further notice. Protein concentration was assessed with a Pierce BCA protein assay kit (ThermoFisher Scientist, 23225). Thirty micrograms of protein was mixed with loading buffer (final concentrations: glycerol 10%, DTT 0.1%, SDS 2%, Tris pH 6.8 0.062 M) and then loaded and run onto an SDS-PAGE gel (Bolt 4%–12% Bis-Tris, Invitrogen). Proteins were transferred onto a polyvinylidene difluoride (PVDF) membrane (Merck Millipore, IPVH00010) at 4°C overnight. Membranes were then incubated in blocking solution (TBS 1×, 0.1% Tween-20 supplemented with 5% milk) for 2 h. Membranes were rinsed with TBS 1×, 0.1% Tween-20 and incubated with an antibody against EZH2 (Cell Signaling Techonology, 5246S, 1:1,000) or ICAM-1 (biotechne, BBA3, 2 µg/mL) or ATF2 (biotechne, AF4455, 1 µg/mL) or GAPDH (Cell Signaling Techonology, 97166S, 1:1,000) or ARL5b (Novusbio, NBP2-01669, 1:1,000) or Clathrin (BD Biosciences, 610500, 1:1,000) diluted in the blocking solution overnight with agitation. The membrane was further washed and incubated with the anti-rabbit IgG HRP-coupled secondary antibody (Jackson Immunoresearch, 711-035-152) in blocking buffer for 45 min. Detection was performed using Pierce ECL Western Blotting Substrate (ThermoFisher Scientist, 32106) and bands imaged by Fusion (Vilber Lourmat) and quantified in ImageJ. GAPDH and Clathrin were used as loading controls.

### Statistics

Statistical tests were performed using Graphpad Prism version 9 software. All statistical tests are listed in the figure legends and significance is indicated as follows: *P < 0.05; **P < 0.01; ***P < 0.001.

## RESULTS

### HRV16 does not replicate in hMDMs but triggers interferon and pro-inflammatory cytokine responses

**Fig 1:**
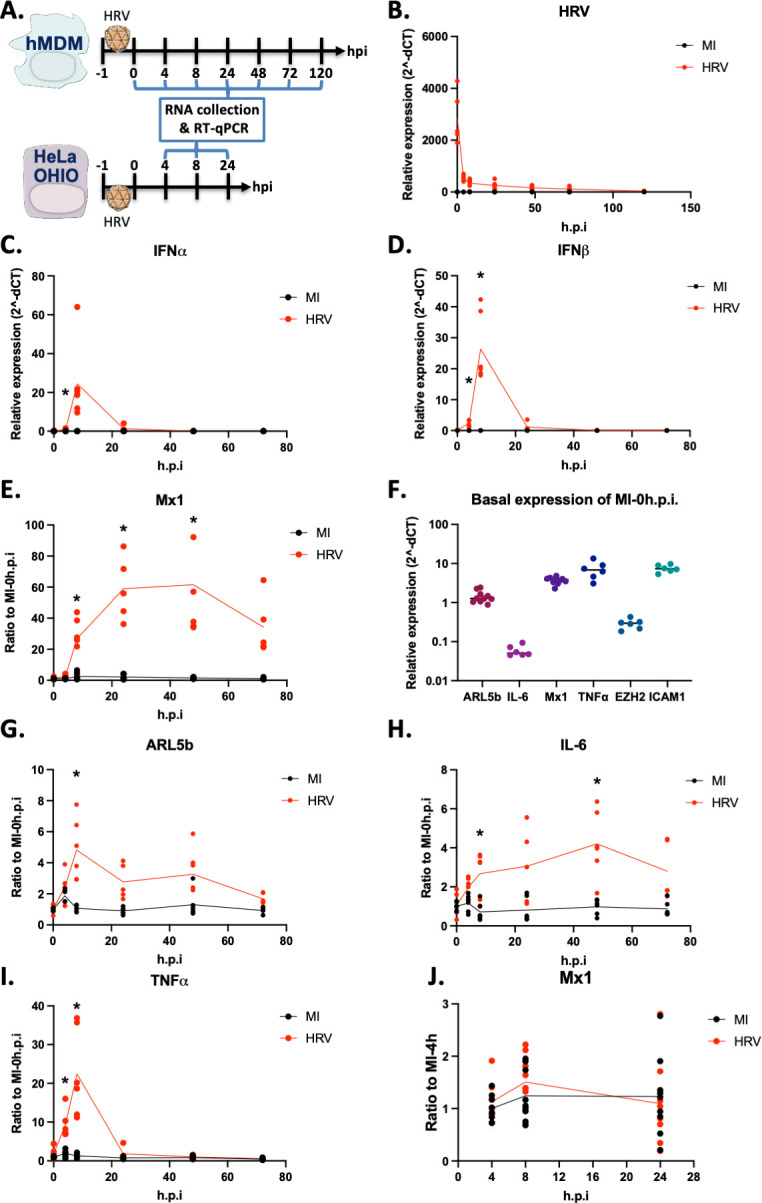
HRV16 does not replicate in hMDMs but triggers an interferon and pro-inflammatory response but not in HeLa OHIO. (**A**) Schematic representation of the experimental timelines. (**B–I**) hMDMs were exposed to 1 × 10^7^ TCID50/mL of HRV16 or mock infected (MI) for 1 h at RT. After 1 h of exposure, cells were washed. Then, cells were lysed at the indicated time points, RNA was extracted, and RT-qPCR was performed. mRNA expressions are presented for (**B**) HRV as relative expression to the house keeping gene; (**C**) IFNα as relative expression to the house keeping gene; (**D**) IFNβ as relative expression to the house keeping gene; (**E**) Mx1 as ratio to the MI at 0 h post infection (h.p.i); (**F**) Mx1, ARL5b, IL-6, TNFα, EZH2, and ICAM-1 as relative expression to the house keeping gene; (**G**) ARL5b as ratio to the MI at 0 h post infection (h.p.i); (**H**) IL-6 as ratio to the MI at 0 h post infection (h.p.i); and (**I**) TNFα as ratio to the MI at 0 h post infection (h.p.i). (**J**) HeLa OHIO were infected at 1 × 10^7^ TCID50/mL of HRV16 or mock infected (MI) for 1 h at RT. After 1 h of infection, cells were washed. Then, cells were lysed at the indicated time point, RNA were extracted, and RT-qPCR was performed. Mx1 expression is presented as ratio to MI 4 h post infection (h.p.i). (**B–J**) Data are the mean of at least three experiments performed in duplicates. Two-way ANOVA statistical analyses were performed. *P < 0.05.

To evaluate the potential of HRV16 to elicit an innate immune response in human monocyte-derived macrophages (hMDMs), cells were exposed to 1.10 (7) TCID50/mL of HRV16 for 1 h, and RNA samples were collected at various time points post-infection (Fig. 1A). HRV RNA was detectable immediately at 0 h post-infection (h.p.i., the time following the 1 h exposure to the virus) but rapidly declined (Fig. 1B). Although HRV was still detectable at 120 h.p.i., there was no evidence of replication, as indicated by the absence of an increase in the quantity of viral RNA post-infection. Notably, IFN-α and IFN-β became detectable between 4 and 24 h.p.i., peaking at approximately 25-fold induction at 8 h.p.i., suggesting a robust type I IFN response (Fig. 1C and D). This IFN induction was followed by the activation of interferon-stimulated genes (ISGs), exemplified by a significant increase in Mx1 expression from 8 h.p.i. onward, reaching a maximum 60-fold induction at 24 and 48 h.p.i. (Fig. 1E). The genes with a detectable basal expression were normalized to the MI 0 h.p.i. sample, with the baseline expression of the corresponding genes presented in Fig. 1F. Consistent with our previous study (13), ARL5b exhibited a significant fivefold induction at 8 h.p.i., gradually returning to basal levels by 72 h.p.i. (Fig. 1G). Additionally, while IL-6 displayed a steady increase from 8 to 48 h.p.i., TNF-α expression mirrored the patterns of IFN-α and IFN-β, with a peak at 8 h.p.i. (Fig. 1H and I).

In our earlier research, we demonstrated that in HRV16-permissive cells, specifically the HeLa OHIO cell line, viral replication was associated with a decrease in ARL5b expression (13). To investigate whether this decrease was linked to an immune response in these cells, we analyzed the type I IFN response. Interestingly, IFN-α and IFN-β mRNAs were not detectable at any of the tested time points (data not shown), and consequently, no induction of Mx1 was observed (Fig. 1J).

Collectively, these findings indicate that while HRV16 can enter macrophages, it does not replicate within these cells. However, HRV16 detection induces interferon and pro-inflammatory responses in macrophages.

### ARL5b is not an interferon-stimulated gene

**Fig 2:**
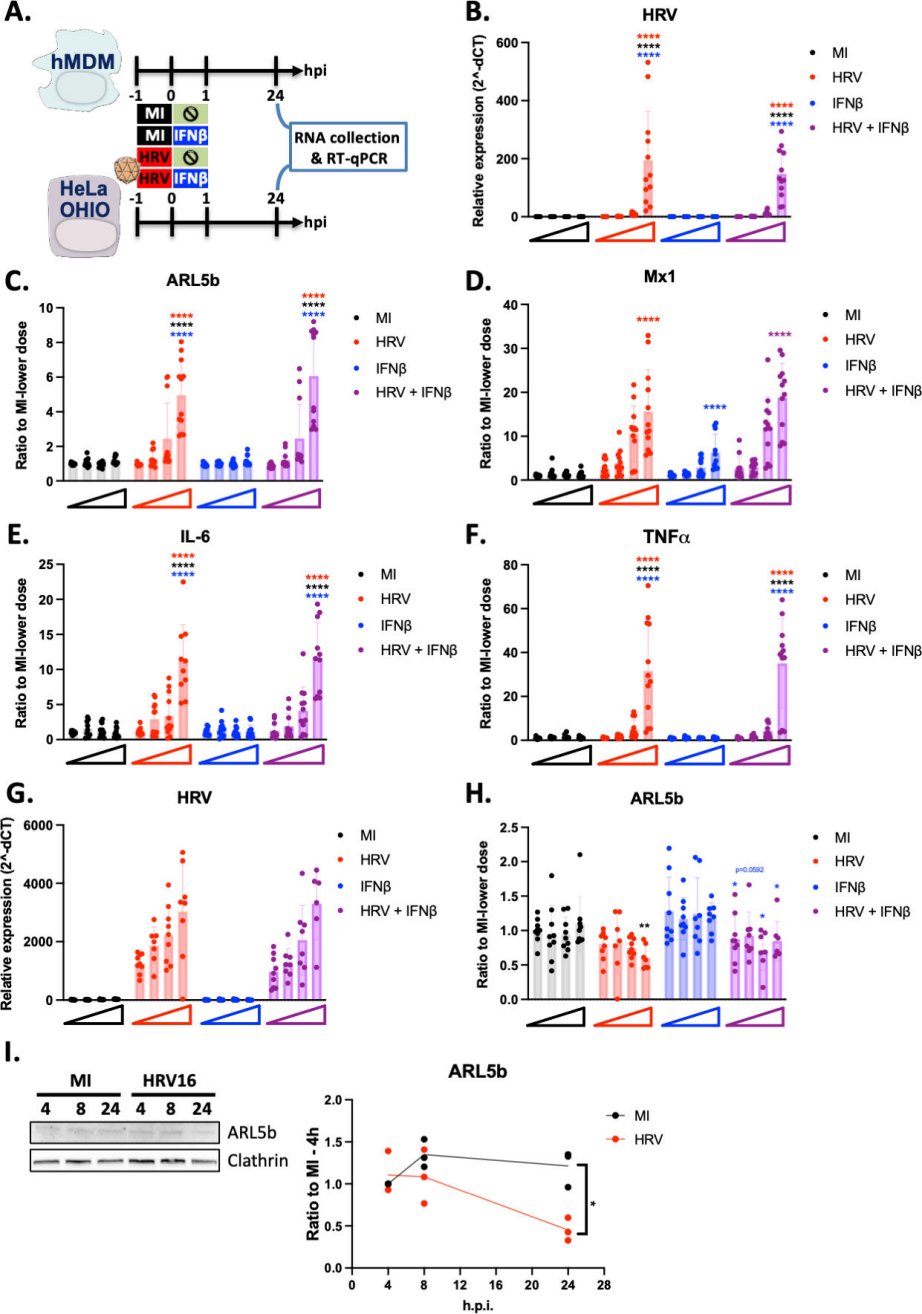
ARL5b is not an interferon stimulated gene. (**A**) Schematic representation of the experimental timelines. (**B–F**) hMDMs were exposed to 1 × 10^4^, 1 × 10^5^, 1 × 10^6^, or 1 × 10^7^ TCID50/mL of HRV16 or mock infected (MI) with the same dilutions, for 1 h at RT. After exposure, cells were treated or not with 1, 10, 100, or 1,000 ng/mL of recombinant IFNβ until cell lysis. Twenty-four hours post exposure, cells were lysed, RNA was extracted, and RT-qPCR was performed. (**B**) HRV, (**C**) ARL5b, (**D**) Mx1, (**E**) IL-6, and (**F**) TNFα mRNA expressions are presented as either (**B**) relative expression to the house keeping gene or (**C–F**) ratio to the MI at the lowest dose. (**G–H**) HeLa OHIO were infected with 1.25 × 10^6^, 2.5 × 10^6^, 5 × 10^6^, or 1 × 10^7^ TCID50/mL of HRV16 or mock infected (MI) with the same dilutions, for 1 h at RT. After infection, cells were treated or not with 1, 10, 100, or 1,000 ng/mL of recombinant IFNβ until cell lysis. Twenty-four hours post infection, cells were lysed, RNA was extracted, and RT-qPCR was performed. (**G**) HRV and (**H**) ARL5b mRNA expressions are presented as either (**G**) relative expression to the house keeping gene or (**H**) ratio to the MI at the lowest dose. (**I**) HeLa OHIO were infected with 1 × 10^7^ TCID50/mL of HRV16 or mock infected (MI) for 1 h at RT. Twenty-four hours post infection, cells were lysed, and proteins were extracted and analysed by immuno-blotting. Band signal was quantified and normalised to Clathrin. ARL5b protein expression is presented as ratio to MI-4h. (**B–I**) Data are the mean ± SD of at least three experiments performed in triplicates. Two-way ANOVA statistical analyses were performed. Red stars indicate statistical differences from HRV16 lowest dose. Black stars indicate statistical differences from all MI conditions. Blue stars indicate statistical differences from IFNβ lowest dose. *P < 0.05; ****P < 0.0001.

ARL5b has previously been proposed to be an interferon-stimulated gene (ISG) (27). To investigate whether the induction of ARL5b in macrophages resulted from HRV16-mediated activation of the interferon response, hMDMs were exposed to increasing doses of HRV and/or increasing doses of recombinant IFN-β (Fig. 2A). It is noteworthy that in the conditions where HRV and recombinant IFN-β were combined, both concentrations were gradually increased. In hMDMs, HRV RNA levels were detectable at exposures of 1 × 10^6^ or 1 × 10 (7) TCID50/mL (Fig. 2B). ARL5b induction was modestly observed at 1 × 10^6^ TCID50/mL (twofold induction) but significantly increased at 1 × 10 (7) TCID50/mL (fivefold induction), both in the presence and absence of IFN-β (Fig. 2C). Notably, even at the highest dose of recombinant IFN-β (1 µg/mL), ARL5b was not induced, nor was there any additional effect of recombinant IFN-β in the presence of HRV16 (Fig. 2C). As a control, Mx1, a well-known ISG, was significantly upregulated with the highest dose of HRV16 (18-fold induction) and recombinant IFN-β (sevenfold induction) (Fig. 2D). This indicates that while the IFN-β stimulation was modest, it was effective under the experimental conditions used. Similar results to ARL5b were obtained for IL-6 and TNFα (Fig. 2E and F).

No IFN response was detected in the presence of HRV16 in HeLa OHIO cells, and recombinant IFN-β had no impact on HRV RNA levels (Fig. 2G). A HRV16-mediated decrease in ARL5b expression was observed only at 1 × 10^7^ TCID50/mL, resulting in a 0.51-fold decrease when comparing “MI” to “HRV” and a 0.60-fold decrease when comparing “recombinant IFN-β” to “HRV +recombinant IFN-β” conditions (Fig. 2H). ARL5b decrease was confirmed at the protein level in HRV16 infected cells compared to MI cells (Fig. 2I). Although not significant, recombinant IFN-β induced a slight increase in ARL5b expression (Fig. 2H). Thus, even though ARL5b expression was partially decreased when comparing the “recombinant IFN-β” condition to the “HRV +recombinant IFN-β” condition, no significant differences were observed between the “MI” and the “HRV + recombinant IFN-β” conditions.

Collectively, our findings indicate that ARL5b is not an ISG in either hMDMs or HeLa OHIO cells. Therefore, the HRV16-mediated upregulation of ARL5b is dependent on one or several signaling pathway(s) activated upon exposure of macrophages to HRV16.

### ARL5b modulation and immune induction by HRV16 is dependent on ICAM-1

**Fig 3:**
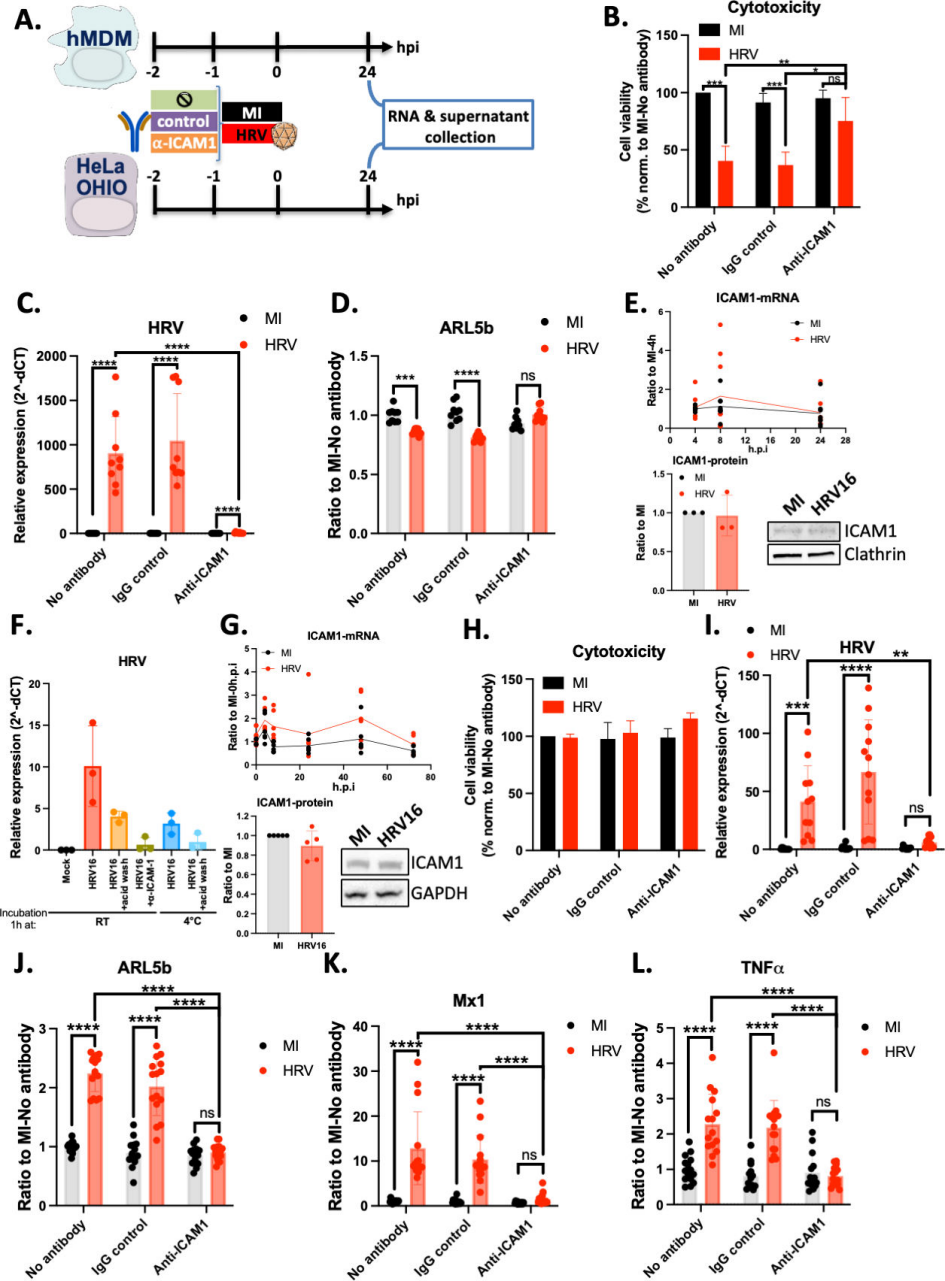
ARL5b regulation in hMDMs and HeLa OHIO is dependent of the viral entry receptor ICAM-1. (**A**) Schematic representation of the experimental timelines of panels B, C, G, H, I, and J. Experimental timelines for panels C and E can be found in Fig. 1A. (**B–D**) HeLa OHIO were treated for 1 h with nothing, a control antibody, or an antibody against ICAM-1 at 37°C. Afterward, cells were infected at 1 × 10^7^ TCID50/mL of HRV16 or mock infected (MI) for 1 h at RT. Twenty-four hours post infection, supernatants were collected, and cells were lysed. (**B**) Cytotoxicity was measured using LDH release quantification. (**C and D**) RNAs were extracted, and RT-qPCR was performed. (**C**) HRV and (**D**) ARL5b expressions are presented as either (**C**) relative expression to the house keeping gene or (**D**) ratio to the MI-no antibody condition. (**E**) HeLa OHIO were infected at 1 × 10^7^ TCID50/mL of HRV16 or MI for 1 h at RT. After 1 h of infection, cells were washed. Then, cells were lysed at the indicated time point, RNA was extracted, and RT-qPCR was performed, or cells were lysed at 24 h post infection; proteins were extracted and analysed by immuno-blotting. Band signal was quantified and normalized to the house keeping protein (Clathrin). ICAM-1 mRNA expression is presented as ratio to MI 4 h post infection (h.p.i). ICAM-1 protein expression is presented as ration to MI. (**B–E**) Data are presented as the mean ± SD of three experiments performed in duplicates. (**F**) hMDMs were treated for 1 h with nothing, or an antibody against ICAM-1 at 37°C. Afterward, cells were exposed to 1 × 10^7^ TCID50/mL of HRV16 or mock infected (MI) for 1 h at either RT or 4°C. After 1 h of exposure, cells were washed either with PBS or with an acid buffer. Cells were lysed right away, RNA was extracted, and RT-qPCR was performed. HRV expression is presented as relative expression to the house keeping gene. Data are presented as the mean ± SD of three experiments. (**G**) hMDMs were exposed to 1 × 10^7^ TCID50/mL of HRV16 or mock infected (MI) for 1 h at RT. After 1 h of exposure, cells were washed. Then, cells were lysed at the indicated time point, RNA was extracted, and RT-qPCR was performed, or cells were lysed at 24 h post infection; proteins were extracted and analysed by immuno-blotting. Band signal was quantified and normalized to the house keeping protein (GAPDH). ICAM-1 mRNA expression is presented as ratio to MI 0 h post infection (h.p.i). Data are the mean of three experiments performed in duplicates. ICAM-1 protein expression is presented as ratio to MI. Data are the mean of five experiments. (**H–L**) hMDMs were treated for 1 h with nothing, a control antibody, or an antibody against ICAM-1 at 37°C. Afterward, cells were exposed to 1 × 10^7^ TCID50/mL of HRV16 or mock infected (MI) for 1 h at RT. Twenty-four hours post exposure, supernatants were collected, and cells were lysed. (**H**) Cytotoxicity was measured using LDH release quantification. (**I–L**) RNAs were extracted, and RT-qPCR was performed. (**I**) HRV, (**J**) ARL5b, (**K**) Mx1, and (**L**) TNFα mRNA expressions are presented as either (**I**) relative expression to the house keeping gene or (**J–L**) ratio to the MI-no antibody condition. (**H–L**) Data are presented as the mean ± SD of four experiments performed in triplicates. (**B–D, H–L**) Two-way ANOVA statistical analyses were performed. **P < 0.01; ***P < 0.001; ****P < 0.0001; ns, not significative.

To investigate the signaling pathways activated upon exposure to HRV16, we first examined the potential involvement of ICAM-1, the cell surface receptor that mediates HRV16 entry in permissive cells. A neutralizing antibody against ICAM-1 was used to assess its role in ARL5b modulation (Fig. 3A).

In HeLa OHIO cells, we first assessed the cytotoxicity of the treatment. HRV16 replication in HeLa OHIO cells induced significant cytotoxicity, as evidenced by a 60% decrease in cell viability (Fig. 3B). However, inhibition of ICAM-1 prevented the cytotoxic effect of HRV16 replication, suggesting an inhibition of viral entry in these cells (Fig. 3B). Indeed, the inhibition of ICAM-1 fully prevented viral replication in HeLa OHIO (Fig. 3C). In that case, the 0.14- to 0.19-fold HRV16-mediated inhibition of ARL5b was abolished, indicating that viral entry and/or replication is crucial for the observed phenotype (Fig. 3D). Importantly, ICAM-1 mRNA and proteins levels were unaffected by HRV16 infection (Fig. 3E).

Since HRV16 does not replicate in hMDMs, we first explored whether HRV16 binding to its receptor and/or viral entry, as indicated by the presence of viral RNA (Fig. 1B), was dependent on ICAM-1. HRV16 was detectable in hMDMs after 1 h of contact at RT (Fig. 3F). To determine if the detected viral genome resulted from the entry of virions into the cells or merely virus binding to the cell surface, hMDMs were washed with an acidic buffer to remove all non-internalized virions after the 1 h-contact with the virus at RT. The same analysis was performed after incubating the cells with HRV16 for 1 h at 4°C to assess acid wash efficiency. HRV16 RNA levels decreased roughly by half with the acid wash, indicating that at least part of the virions did enter the cells. Significantly, the anti-ICAM-1 antibody prevented HRV16 internalization, indicating that ICAM-1 serves as a receptor for HRV16 in hMDMs (Fig. 3F). At 4°C, the levels of HRV16 detected were decreased, highlighting endocytosis-dependent entry (Fig. 3F). Notably, ICAM-1 mRNA and protein levels were not modulated by HRV16 (Fig. 3G). Notably, cells treated with the ICAM-1 neutralizing antibody or control IgG exhibited no cytotoxicity, nor did exposure to HRV16 (Fig. 3H). Prevention of HRV16 entry was confirmed at 24 h.p.i., as no HRV16 RNA was detected in anti-ICAM-1 treated cells (Fig. 3I). Interestingly, ICAM-1 inhibition prevented HRV16-mediated induction of ARL5b, Mx1, and TNFα (Fig. 3J through L).

In summary, our results demonstrate that the modulation of ARL5b is dependent on ICAM-1/viral entry in both hMDMs and HeLa OHIO cells. Additionally, immune induction in hMDMs also relies on ICAM-1/viral entry.

### HRV16 activation of ARL5b and immune response in hMDMs is dependent on PKR

To further explore the signaling pathways activated by HRV16 following ICAM-1 binding and viral entry, we investigated the role of Protein Kinase R (PKR). PKR is known to participate in various signaling cascades, including those involved in pathogen detection and the interferon (IFN) response (21, 28). We evaluated whether PKR could be involved in HRV16-mediated induction of an immune response.

**Fig 4:**
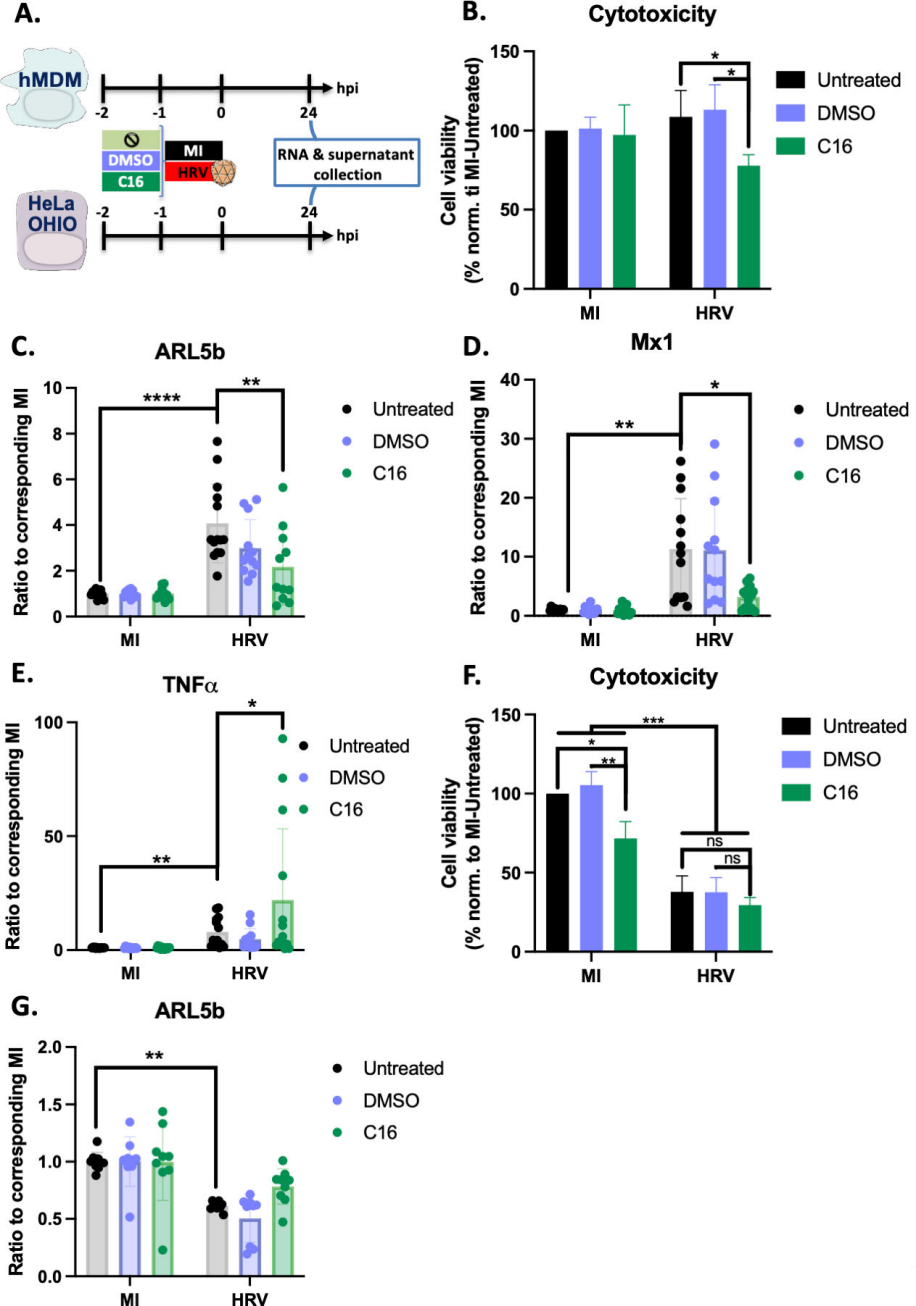
ARL5b induction in hMDMs is dependent on PKR. (**A**) Schematic representation of the experimental timelines. (**B–E**) hMDMs were treated for 1 h with nothing, DMSO, or 50 µM of C16 (PKR inhibitor) at 37°C. Afterward, cells were exposed to 1 × 10^7^ TCID50/mL of HRV16 or mock infected (MI) for 1 h at RT. Twenty-four hours post exposure, supernatants were collected, and cells were lysed. (**B**) Cytotoxicity was measured using LDH release quantification. (**C–E**) RNA was extracted, and RT-qPCR was performed. (**C**) ARL5b, (**D**) Mx1, and (**E**) TNFα expressions are presented as ratio to the corresponding MI conditions. (**F and G**) HeLa OHIO were treated for 1 h with nothing, DMSO, or 50 µM of C16 (PKR inhibitor) at 37°C. Afterward, cells were infected at 1 × 10^7^ TCID50/mL of HRV16 or mock infected (MI) for 1 h at RT. Twenty-four hours post infection, supernatants were collected, and cells were lysed. (**F**) Cytotoxicity was measured using LDH release quantification. (**G**) RNA was extracted, and RT-qPCR was performed. ARL5b expression is presented as ratio to the corresponding MI conditions. (**B–G**) Data are presented as mean ± SD of at least three experiments performed in triplicates. Two-way ANOVA statistical analyses were performed. *P < 0.05; **P < 0.01; ****P < 0.0001; ns, not significative.

Cells were treated with C16, an inhibitor of PKR, an equivalent amount of DMSO as a control, or left untreated (Fig. 4A). C16 treatment resulted in a 25% decrease in hMDM viability, indicating a cytotoxic effect (Fig. 4B). HRV16 induced a two- to eightfold increase of ARL5b, which was reduced by an average 54% following C16 treatment. This suggests that PKR is at least partially involved in the upregulation of ARL5b (Fig. 4C). In addition, PKR inhibition also prevented the HRV16-induced upregulation of Mx1 (Fig. 4D). In some donors, C16 treatment unexpectedly enhanced TNF induction by HRV16 (Fig. 4E), indicating a complex interplay of signaling pathways. These findings indicate that PKR is involved in the signaling pathways activated by HRV16 to induce ARL5b expression.

Similar experiments were conducted on HeLa OHIO cells to determine if PKR plays a role in the inhibition of ARL5b expression in these cells. Cell cytotoxicity assessment showed that C16 alone exhibited a 30% cytotoxic effect on HeLa OHIO (condition “MI-C16”, Fig. 4F). Upon infection with HRV, the exposure to C16 showed no significant additive toxicity as compared to the viral replication itself (Fig. 4F). Moreover, HRV-mediated ARL5b decrease was not significantly rescued in C16-treated cells compared to the control conditions (Fig. 4G). These data suggest that in HeLa OHIO cells, the inhibition of ARL5b is independent of PKR signaling.

The contrasting results between hMDMs and HeLa OHIO cells underscore potential cell-type-specific differences in the signaling pathways activated by HRV16.

### HRV16 activation of ARL5b and Mx1 in hMDMs is dependent on ATF2

To gain further insight into the signaling induced by HRV16 in hMDMs, we focused on transcription factors known to be activated by PKR, including c-Jun, ATF2, and NF-κB (23). ATF2, which has previously been shown to be activated by HRV16 (16), was of particular interest in deciphering the mechanisms involved in the HRV16 and was of particular interest in understanding the mechanisms underlying HRV16-mediated induction of ARL5b.

**Fig 5:**
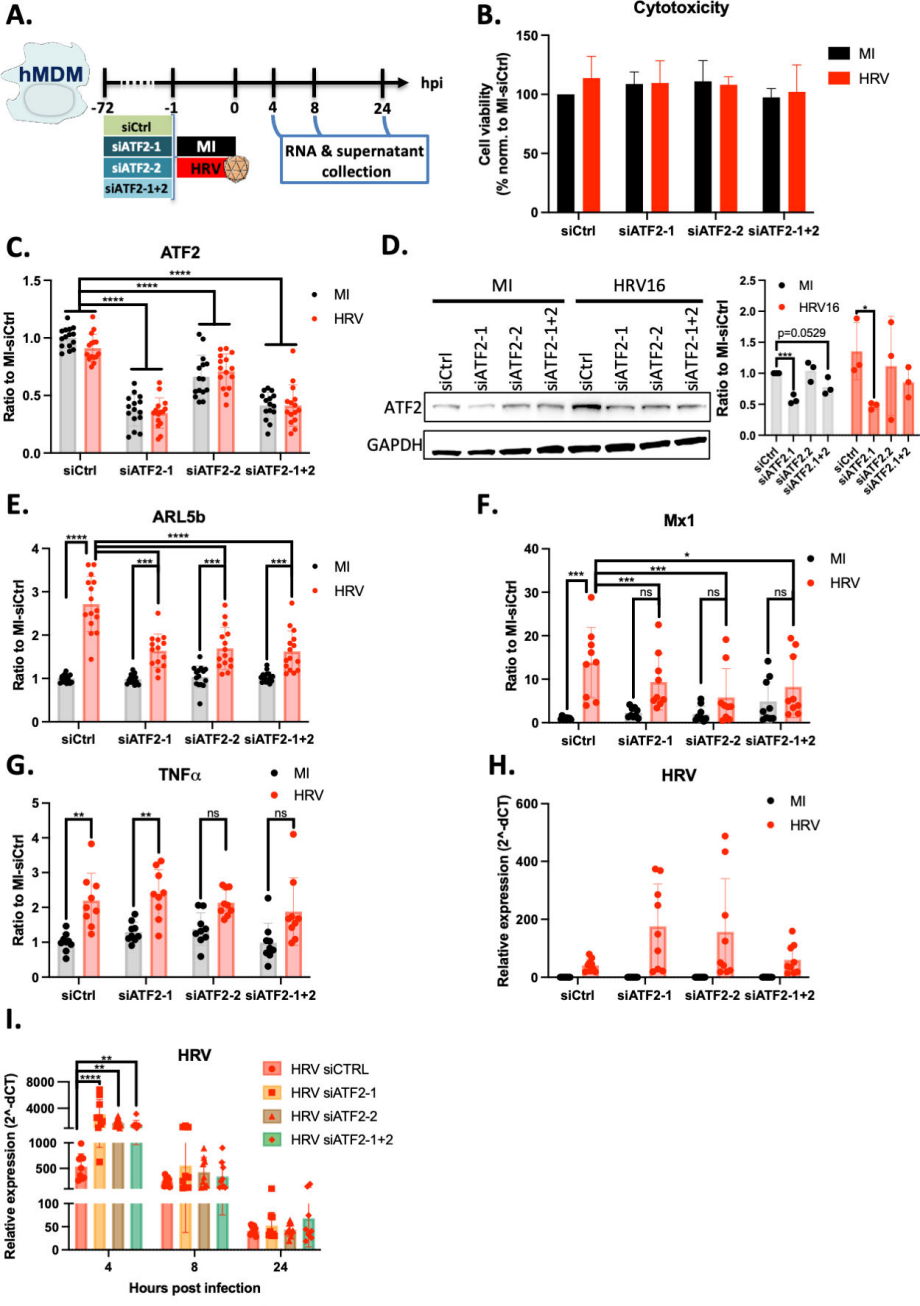
ARL5b induction in hMDMs is dependent on ATF2. (**A**) Schematic representation of the experimental timelines. (**B–H**) hMDMs were transfected with 100 nM of si RNA control (siCtrl) or siRNA against ATF2 (siATF2-1 or si ATF2-2), or a combination of siATF2 (each at 50 nM). 72 h post transfection, cells were exposed to 1 × 10^7^ TCID50/mL of HRV16 or mock infected (MI) for 1 h at RT. Twenty-four hours post exposure, supernatants were collected, and cells were lysed, (**B**) Cytotoxicity was measured using LDH release quantification. (**C, E–H**) RNAs were extracted, and RT-qPCR was performed. (**C**) ATF2, (**E**) ARL5b, (**F**) Mx1, (**G**) TNFα, and (**H**) HRV expressions are presented as either (**C, E–G**) ratio to the corresponding MI-siCtrl condition or (**H**) relative expression normalized to the house keeping gene. (**D**) Proteins were extracted and analyzed by immuno-blotting. The indicated proteins were detected. Band signal was quantified and normalized to the house keeping protein (GAPDH). (**I**) hMDMs were transfected with 100 nM of si RNA control (siCtrl) or siRNA against ATF2 (siATF2-1 or si ATF2-2), or a combination of siATF2 (each at 50 nM). Seventy-two hours post transfection, cells were exposed to 1 × 10^7^ TCID50/mL of HRV16 or mock infected (MI) for 1 h at RT. 4 h, 8 h, or 24 h post exposure, cells were lysed, RNAs were extracted, and RT-qPCR was performed. HRV expression is presented as relative expression normalised to the house keeping gene. (**B–I**) Data are presented as mean ± SD of at least three experiments performed in triplicates. Two-way ANOVA statistical analyses were performed. *P < 0.05; **P < 0.01; ****P < 0.0001.

ATF2 was selectively depleted using siRNAs in hMDMs (Fig. 5A). Treatment with siRNAs and ATF2 depletion showed no cytotoxicity on hMDMs (Fig. 5B). Compared to the control siRNA (siCtrl), siATF2-1 reduced ATF2 mRNA expression by 60%, observed in both the siATF2-1 and siATF2-1 + 2 conditions, while siATF2-2 depleted ATF2 by 30% (Fig. 5C). At the protein level, ATF2 was depleted by 50% with siATF2-1, whereas siATF2-2 did not lead to significant protein depletion at the tested time point (Fig. 5D). In the siATF2-1 + 2 condition, ATF2 was partially depleted (around 25%) (Fig. 5D). In the siCtrl condition, a 2.7-fold increase of ARL5b was observed in presence of HRV16, depletion of ATF2 by siRNAs resulted in 1.6-fold increase of ARL5b induction only (Fig. 5E). Thus, ATF2 depletion led to a 30% decrease in ARL5b induction, highlighting a role for ATF2 in HRV16-mediated ARL5b induction (Fig. 5E). Similar effects were observed for Mx1, whereas ATF2 played no significant role in TNFα induction (Fig. 5F and G). Surprisingly, HRV16 RNA levels increased upon ATF2 depletion in some experiments (Fig. 5H). The increase of HRV RNA expression upon ATF2 silencing suggests two possibilities: (i) HRV may be able to replicate in macrophages in the absence of ATF2 or (ii) the elimination of HRV RNA in macrophages is slowed down by the absence of ATF2. To explore this mechanism, HRV RNA expression was analyzed kinetically after ATF2 depletion. HRV RNA levels were strongly increased at 4 h post exposure in all tested donors, suggesting that the absence of ATF2 may favor the initial accumulation of HRV in hMDMs (Fig. 5I). However, over time, an overall decrease of HRV RNA levels was observed, indicating that HRV is not able to replicate in the hMDMs, even upon ATF2 silencing.

In summary, these results collectively indicate that HRV16 induces ARL5b expression through an ICAM-1/PKR/ATF2 signaling axis in hMDMs.

### HRV16 epigenetically regulates ARL5b promoter

**Fig 6:**
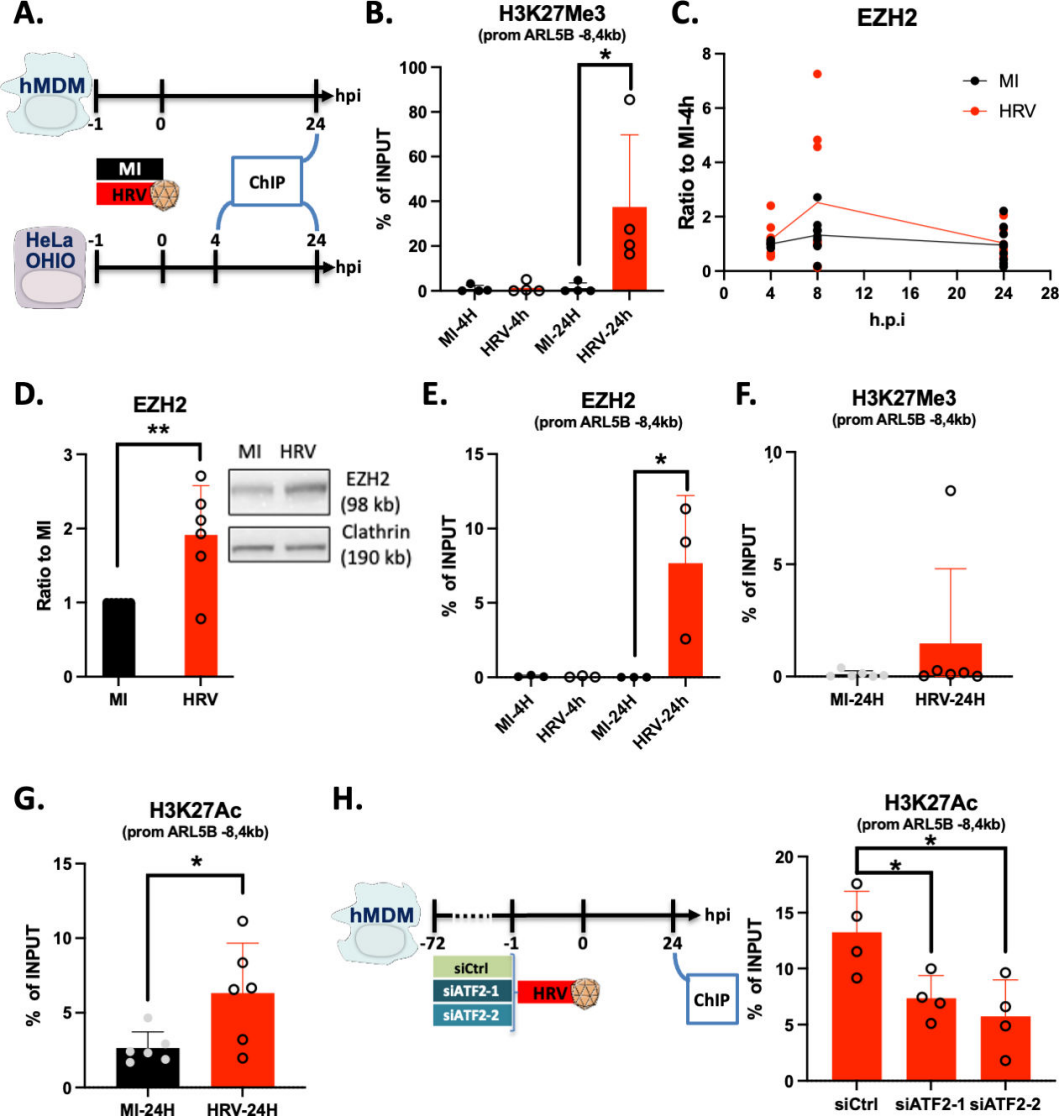
ARL5b promoter is epigenetically regulated upon HRV16 exposure or infection. (**A**) Schematic representation of the experimental timelines for panels B, E, F, and G. Timeline for panel C can be found in Fig. 1A. (**B**) HeLa OHIO were infected at 1 × 10^7^ TCID50/mL of HRV16 or mock infected (MI) for 1 h at RT. 4 h or 24 h post infection, DNA and proteins were crosslinked, DNA was immunoprecipitated using an antibody against H3K27Me3, and qPCR was performed. The enrichment of ARL5b promoter is presented as the percentage of the total input. (**C**) HeLa OHIO were infected at 1 × 10^7^ TCID50/mL of HRV16 or mock infected (MI) for 1 h at RT. After 1 h of infection, cells were washed. Then, cells were lysed at the indicated time point, RNA was extracted, and RT-qPCR was performed. EZH2 expression is presented as ratio to MI 4 h post infection (h.p.i). (**D**) HeLa OHIO were infected at 1 × 10^7^ TCID50/mL of HRV16 or mock infected (MI) for 1 h at RT. Twenty-four hours post infection, cells were lysed, proteins were extracted, and immunoblotting using an antibody against EZH2 was performed. Representative image of immunoblots obtained. Quantification of six independent experiments. Data are represented as ratio to the MI condition. (**E**) HeLa OHIO were infected at 1 × 10^7^ TCID50/mL of HRV16 or mock infected (MI) for 1 h at RT. 4 h or 24 h post infection, DNA and proteins were crosslinked, DNA was immunoprecipitated using an antibody against EZH2, and qPCR was performed. The enrichment of ARL5b promoter is presented as the percentage of the total input. (**F and G**) hMDMs were exposed to 1 × 10^7^ TCID50/mL of HRV16 or mock infected (MI) for 1 h at RT. Twenty-four hours post exposure, DNA and proteins were crosslinked, DNA was immunoprecipitated using an antibody against (**F**) H3K27Me3 or (**G**) H3K27Ac, and qPCR was performed. The enrichment of ARL5b promoter is presented as the percentage of the total input. (**H**) hMDMs were transfected with 100 nM of si RNA control (siCtrl) or siRNA against ATF2 (siATF2-1 or si ATF2-2). Seventy-two hours post transfection, cells were exposed to 1 × 10^7^ TCID50/mL of HRV16 or mock infected (MI) for 1 h at RT. Twenty-four hours post exposure, DNA and proteins were crosslinked, DNA was immunoprecipitated using an antibody against H3K27Ac, and qPCR was performed. The schematic representation of the experimental timeline is indicated on the left. The enrichment of ARL5b promoter is presented as the percentage of the total input (on the right). (**B, D–H**) Data are represented as the mean ± SD of at least four experiments. Student *t*-test statistical analyses were performed. *P < 0.05; **P < 0.01. (**C**) Data are the mean of at least three experiments performed in duplicates. Two-way ANOVA statistical analyses were performed. **P < 0.01.

Finally, we aimed to investigate whether ARL5b expression is regulated at the epigenetic level. To explore this, HeLa OHIO cells were exposed to the virus, and at various time points post-infection, DNA and proteins were crosslinked and analyzed by chromatin immunoprecipitation (ChIP) (Fig. 6A). Given that ARL5b expression is repressed by HRV16 in these cells, we examined whether the repressive epigenetic mark H3K27Me3 exhibited differential enrichment on the ARL5b promoter. At 24 h.p.i., H3K27Me3 was found to be enriched on the ARL5b promoter in HRV16-treated cells compared to MI-treated cells or the 4 h.p.i. conditions (Fig. 6B). This increase in H3K27Me3 could potentially explain the observed repression of the gene. The trimethylation of lysine 27 of histone 3 is mediated by the methyltransferase EZH2 (26). Thus, we then assessed whether HRV16 infection in HeLa OHIO could modify EZH2 expression. While there was no significant modulation of EZH2 at the mRNA level (Fig. 6C), protein expression increased by twofold, as demonstrated by immunoblotting (Fig. 6D). Concurrently, EZH2 recruitment to the ARL5b promoter was increased at 24 h.p.i. (Fig. 6E).

Next, we examined if similar epigenetic modulations of ARL5b promoter could be observed in macrophages (Fig. 6A). H3K27Me3 presence on the ARL5b promoter did not differ between HRV16 and MI-challenged hMDMs (Fig. 6F). Since ARL5b expression is increased by HRV16 in hMDMs, we studied the level of the activating epigenetic mark H3K27Ac on the ARL5b promoter. H3K27Ac was twice as much present on the ARL5b promoter in HRV16-challenged cells than in the MI condition (Fig. 6G). Finally, to investigate if ATF2 activation could be linked to the observed increase in H3K27Ac (29), hMDMs were depleted for ATF2, exposed to HRV16, and analyzed by ChIP. The results showed that ATF2 depletion was associated with a decrease in H3K27Ac on the ARL5b promoter (Fig. 6H).

In summary, these data demonstrate that HRV16 induces epigenetic modifications on the promoter of ARL5b, either associated with repression in HeLa OHIO or with activation in hMDMs. The HRV16-mediated increase in H3K27Ac on the ARL5b promoter in hMDMs is dependent on ATF2.

## DISCUSSION

In this study, we demonstrated that HRV16 does not replicate in hMDMs but induces type I interferon and pro-inflammatory cytokine responses. Our focus was to understand the regulation of ARL5b, a protein we identified as being involved in the impairment of phagosome maturation (13). As observed previously, ARL5b is upregulated in hMDMs exposed to HRV16. This upregulation is dependent on the ICAM-1/PKR/ATF2 signaling axis and the subsequent epigenetic activation of the ARL5b promoter. In contrast, the HRV16-permissive HeLa OHIO cells do not exhibit an immune response upon infection. In these cells, ARL5b, which acts as a viral restriction factor in these cells, is downregulated in an ICAM-1-dependent manner, but independently of PKR signaling. This downregulation is associated with an EZH2-dependent repression of the ARL5b promoter.

In the current study, we revisited the question of HRV16 replication in macrophages, considering previous findings by Laza-Stanca et al. and our own work (9, 20). Laza-Stanca et al. demonstrated HRV16 replication in macrophage-like cells derived from the monocytic THP1 cell line. However, consistent with our data, they observed no increase in HRV16 viral load in hMDMs, suggesting that the virus may not replicate in these cells. Discrepancies between the THP1-derived macrophages and hMDMs cellular models could be attributed to the fact that THP1 cells are a transformed cell line, which might affect viral detection or create conditions favorable for viral replication. Interestingly, despite the lack of replication or a complete viral cycle in hMDMs, HRV16 can induce epigenetic modifications and profoundly disrupt macrophage functions. This indicates that the virus can hijack cellular functions even in cells non-permissive to replication. Additionally, in our previous study, UV-inactivated HRV16 could not induce ARL5b expression, which was suggested to be due to impairment of the viral genome or its replication (13). Our current results further suggest that UV inactivation of HRV16 may prevent ARL5b induction by impairing viral binding to ICAM-1, decapsidation inside macrophages, or detection by cytosolic sensors.

Several immune sensors could be involved in the recognition of HRV16, especially those detecting viral RNA. Among these sensors, we have shown that PKR (21) is involved in the detection of HRV16. Nevertheless, the contribution of other immune sensors cannot be excluded. As PKR is a cytosolic sensor, we hypothesize that the HRV16 genome is released into the cytoplasm of macrophages, where it can be detected by PKR. It is also possible that, in macrophages, the binding of HRV16 to ICAM-1 triggers an unknown signalling, resulting in PKR activation.

Our study also revealed that HRV16-induced modulation of ARL5b expression is associated with epigenetic modifications, with a positive effect in macrophages and a negative effect in HeLa OHIO cells. The association of the positive epigenetic mark H3K27Ac with the ARL5b promoter in macrophages was linked to the activation of the transcription factor ATF2. Notably, ATF2 has a predicted binding site on the ARL5b promoter in the region where H3K27Ac was increased. ATF2 has also been described as having an acetyltransferase activity (29). Altogether, this leads to the hypothesis that activated ATF2 may bind to the ARL5b promoter, leading to promoter acetylation and, consequently, the induction of ARL5b expression.

Interestingly, in HeLa OHIO cells, the opposite effect is observed, with upregulation of the repressive mark H3K27Me3 on the ARL5b promoter, which is associated with increased recruitment of the methyltransferase EZH2. An increase in total EZH2 expression is also observed in these cells. Given that PKR signaling is not involved in ARL5b regulation in these cells, it is possible that either the viral replication or one or more viral protein is involved in upregulating EZH2 and the subsequent repression of ARL5b promoter.

Furthermore, our study confirms that HRV16 can induce type I interferon and pro-inflammatory immune responses in macrophages, in line with previously published data (12, 16–18, 30). Importantly, our findings provide the first evidence that HRV16-mediated induction of Mx1 and TNFα in macrophages is dependent on the cellular receptor for viral entry, ICAM-1. Whether binding to the receptor and intracellular signaling alone or viral entry into macrophages is necessary for activating the immune response is currently unknown. ICAM-1 expression has previously been shown to be upregulated in upper airway epithelial cells upon HRV infection (31). In the cell models we used (hMDMs and HeLa OHIO), no modulation in ICAM-1 expression was observed upon HRV16 infection. These differences might be due to the fact that the increase of ICAM-1 expression upon HRV infection is specific to upper airway epithelial cells and/or specific to HRV14 and HRV39, as used by Winther and colleagues.

Finally, our study revealed that while the induction of ARL5b expression and the interferon response in macrophages depended partly on PKR and ATF2 signaling, this was not the case for TNFα. TNFα has been previously demonstrated to be activated by HRV through an NF-κB-dependent mechanism, which may also be applicable in our model (20). The partial inhibition of ARL5b induction observed upon PKR or ATF2 modulation suggests that HRV16 can induce additional intracellular sensing and signaling pathways in macrophages. Importantly, the activation of the PKR/ATF2 axis in macrophages could potentially trigger the expression of other genes involved in viral regulation as well as the impairment of macrophage functions, similar to the effect observed on ARL5b.

**Fig 7:**
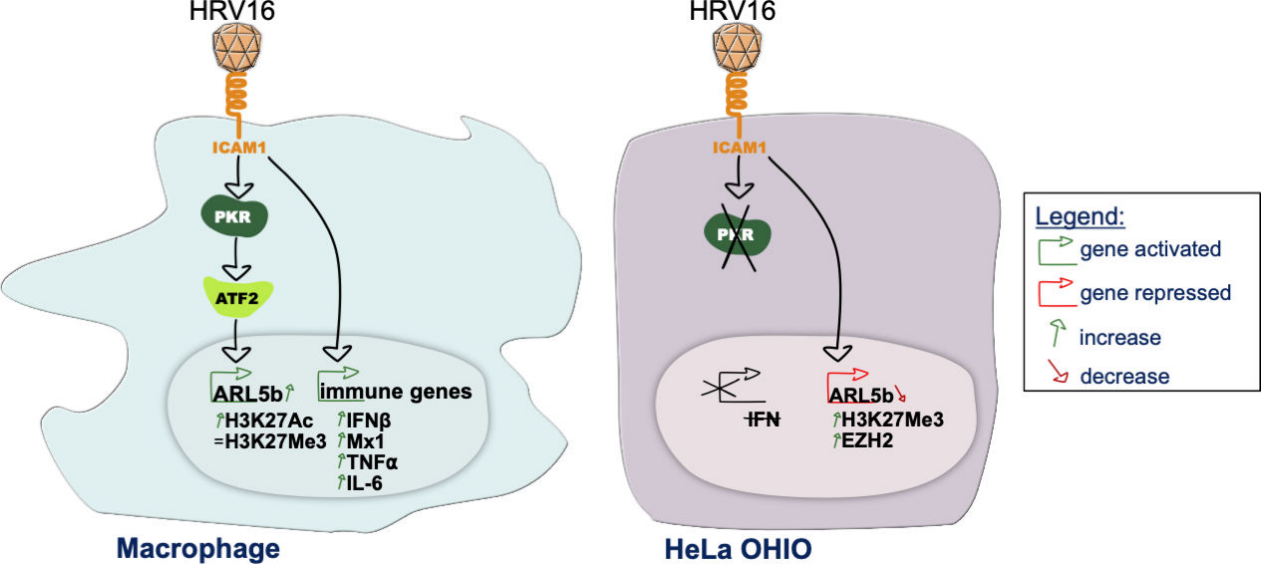
Graphical representation of the finding of this study. HRV16 enters macrophages and HeLa OHIO in an ICAM-1-dependent manner. In macrophages, HRV16 triggers cell activation and immune genes expression. ARL5b activation is dependent on the activation of PKR and subsequently of the transcription factor ATF2. Activated ATF2 can translocate to the nucleus and activate gene expression. This is associated with an increase of the positive epigenetic modification H3K27Ac on ARL5b promoter. In HeLa OHIO, HRV16 does not induce interferon and ARL5b repression is independent of PKR. ARL5b repression is due to the upregulation of EZH2 and the increase of the repressive epigenetic mark H3K27ME3 on ARL5b promoter.

In summary, our findings indicate that HRV16 does not replicate in macrophages, yet it elicits type I interferon and pro-inflammatory responses. We demonstrated that the induction of ARL5b is dependent on an ICAM-1/PKR/ATF2 signaling axis and subsequent epigenetic modifications in macrophages (Fig. 7). In HeLa OHIO cells, the inhibition of the restriction factor ARL5b is dependent on ICAM-1, but not on PKR (Fig. 7). While further exploration is needed to decipher these mechanisms, targeting this signaling pathway could be of interest for patients experiencing disease exacerbations after HRV infection. Therefore, this dual regulation opens avenues for potential targeted treatments to prevent ARL5b induction in macrophages, facilitating effective bacterial clearance.

## Data Availability

The data that support the findings of this study are available from the corresponding author, S.F.-D., upon request.

## SUPPLEMENTAL MATERIAL

Table S1 jvi.01499-24-s0001.xlsx Primer list.

ASM does not own the copyrights to Supplemental Material that may be linked to, or accessed through, an article. The authors have granted ASM a non-exclusive, world-wide license to publish the Supplemental Material files. Please contact the corresponding author directly for reuse.

## References

[1] Jacobs SE, Lamson DM, St George K, Walsh TJ. 2013. Human rhinoviruses. Clin Microbiol Rev 26:135–162. doi:10.1128/CMR.00077-12 23297263 PMC3553670

[2] Bizot E, Bousquet A, Charpié M, Coquelin F, Lefevre S, Le Lorier J, Patin M, Sée P, Sarfati E, Walle S, Visseaux B, Basmaci R. 2021. Rhinovirus: a narrative review on its genetic characteristics, pediatric clinical presentations, and pathogenesis. Front Pediatr 9:643219. doi:10.3389/fped.2021.643219 33829004 PMC8019700

[3] Yang Z, Mitländer H, Vuorinen T, Finotto S. 2021. Mechanism of rhinovirus immunity and asthma. Front Immunol 12:731846. doi:10.3389/fimmu.2021.731846 34691038 PMC8526928

[4] Gern JE, Galagan DM, Jarjour NN, Dick EC, Busse WW. 1997. Detection of rhinovirus RNA in lower airway cells during experimentally induced infection. Am J Respir Crit Care Med 155:1159–1161. doi:10.1164/ajrccm.155.3.9117003 9117003

[5] Wilkinson TMA, Hurst JR, Perera WR, Wilks M, Donaldson GC, Wedzicha JA. 2006. Effect of interactions between lower airway bacterial and rhinoviral infection in exacerbations of COPD. Chest 129:317–324. doi:10.1378/chest.129.2.317 16478847 PMC7094441

[6] Wilkinson TMA, Aris E, Bourne S, Clarke SC, Peeters M, Pascal TG, Schoonbroodt S, Tuck AC, Kim V, Ostridge K, Staples KJ, Williams N, Williams A, Wootton S, Devaster J-M, AERIS Study Group. 2017. A prospective, observational cohort study of the seasonal dynamics of airway pathogens in the aetiology of exacerbations in COPD. Thorax 72:919–927. doi:10.1136/thoraxjnl-2016-209023 28432209 PMC5738531

[7] Bellinghausen C, Rohde GGU, Savelkoul PHM, Wouters EFM, Stassen FRM. 2016. Viral-bacterial interactions in the respiratory tract. J Gen Virol 97:3089–3102. doi:10.1099/jgv.0.000627 27902340

[8] Jubrail J., Kurian N, Niedergang F. 2017. Macrophage phagocytosis cracking the defect code in COPD. Biomed J 40:305–312. doi:10.1016/j.bj.2017.09.004 29433833 PMC6138611

[9] Jubrail J, Africano-Gomez K, Herit F, Mularski A, Bourdoncle P, Oberg L, Israelsson E, Burgel P-R, Mayer G, Cunoosamy DM, Kurian N, Niedergang F. 2020. Arpin is critical for phagocytosis in macrophages and is targeted by human rhinovirus. EMBO Rep 21:e47963. doi:10.15252/embr.201947963 31721415 PMC6945061

[10] Oliver BGG, Lim S, Wark P, Laza-Stanca V, King N, Black JL, Burgess JK, Roth M, Johnston SL. 2008. Rhinovirus exposure impairs immune responses to bacterial products in human alveolar macrophages. Thorax 63:519–525. doi:10.1136/thx.2007.081752 18245149

[11] Belchamber KBR, Singh R, Batista CM, Whyte MK, Dockrell DH, Kilty I, Robinson MJ, Wedzicha JA, Barnes PJ, Donnelly LE, COPD-MAP consortium. 2019. Defective bacterial phagocytosis is associated with dysfunctional mitochondria in COPD macrophages. Eur Respir J 54:1802244. doi:10.1183/13993003.02244-2018 31320451

[12] Finney LJ, Belchamber KBR, Fenwick PS, Kemp SV, Edwards MR, Mallia P, Donaldson G, Johnston SL, Donnelly LE, Wedzicha JA. 2019. Human rhinovirus impairs the innate immune response to bacteria in alveolar macrophages in chronic obstructive pulmonary disease. Am J Respir Crit Care Med 199:1496–1507. doi:10.1164/rccm.201806-1095OC 30562053

[13] Faure-Dupuy S, Jubrail J, Depierre M, Africano-Gomez K, Öberg L, Israelsson E, Thörn K, Delevoye C, Castellano F, Herit F, Guilbert T, Russell DG, Mayer G, Cunoosamy DM, Kurian N, Niedergang F. 2024. ARL5b inhibits human rhinovirus 16 propagation and impairs macrophage-mediated bacterial clearance. EMBO Rep 25:1156–1175. doi:10.1038/s44319-024-00069-x 38332148 PMC10933434

[14] Depierre M, Jacquelin L, Niedergang F. 2023. Phagocytosis, p 286–295. In Bradshaw RA, Hart GW, Stahl PD (ed), Encyclopedia of cell biology, 2nd ed. Academic Press. doi:10.1016/B978-0-12-821618-7.00038-9.

[15] Uribe-Querol E, Rosales C. 2020. Phagocytosis: our current understanding of a universal biological process. Front Immunol 11:1066. doi:10.3389/fimmu.2020.01066 32582172 PMC7280488

[16] Schuler BA, Schreiber MT, Li L, Mokry M, Kingdon ML, Raugi DN, Smith C, Hameister C, Racaniello VR, Hall DJ. 2014. Major and minor group rhinoviruses elicit differential signaling and cytokine responses as a function of receptor-mediated signal transduction. PLoS One 9:e93897. doi:10.1371/journal.pone.0093897 24736642 PMC3988043

[17] Korpi-Steiner NL, Bates ME, Lee W-M, Hall DJ, Bertics PJ. 2006. Human rhinovirus induces robust IP-10 release by monocytic cells, which is independent of viral replication but linked to type I interferon receptor ligation and STAT1 activation. J Leukoc Biol 80:1364–1374. doi:10.1189/jlb.0606412 17020930

[18] Jubrail J, Africano-Gomez K, Herit F, Baturcam E, Mayer G, Cunoosamy DM, Kurian N, Niedergang F. 2018. HRV16 impairs macrophages cytokine response to a secondary bacterial trigger. Front Immunol 9:2908. doi:10.3389/fimmu.2018.02908 30619272 PMC6305396

[19] Warner SM, Wiehler S, Michi AN, Proud D. 2019. Rhinovirus replication and innate immunity in highly differentiated human airway epithelial cells. Respir Res 20:150. doi:10.1186/s12931-019-1120-0 31299975 PMC6626354

[20] Laza-Stanca V, Stanciu LA, Message SD, Edwards MR, Gern JE, Johnston SL. 2006. Rhinovirus replication in human macrophages induces NF-κB-dependent tumor necrosis factor alpha production. J Virol 80:8248–8258. doi:10.1128/JVI.00162-06 16873280 PMC1563804

[21] Cottrell KA, Andrews RJ, Bass BL. 2024. The competitive landscape of the dsRNA world. Mol Cell 84:107–119. doi:10.1016/j.molcel.2023.11.033 38118451 PMC10843539

[22] Gal-Ben-Ari S, Barrera I, Ehrlich M, Rosenblum K. 2018. PKR: a kinase to remember. Front Mol Neurosci 11:480. doi:10.3389/fnmol.2018.00480 30686999 PMC6333748

[23] Kang R, Tang D. 2012. PKR-dependent inflammatory signals. Sci Signal 5:pe47. doi:10.1126/scisignal.2003511 23092889 PMC3656404

[24] Czimmerer Z, Nagy L. 2023. Epigenomic regulation of macrophage polarization: where do the nuclear receptors belong? Immunol Rev 317:152–165. doi:10.1111/imr.13209 37074820 PMC10524119

[25] Locatelli M, Faure-Dupuy S. 2023. Virus hijacking of host epigenetic machinery to impair immune response. J Virol 97:e0065823. doi:10.1128/jvi.00658-23 37656959 PMC10537592

[26] Kim KH, Roberts CWM. 2016. Targeting EZH2 in cancer. Nat Med 22:128–134. doi:10.1038/nm.4036 26845405 PMC4918227

[27] Boppana S, Mindur JE, Balashov KE, Dhib-Jalbut S, Ito K. 2013. Comparison of IFN-β inducible gene expression in primary-progressive and relapsing-remitting multiple sclerosis. J Neuroimmunol 265:68–74. doi:10.1016/j.jneuroim.2013.10.007 24200257

[28] García MA, Gil J, Ventoso I, Guerra S, Domingo E, Rivas C, Esteban M. 2006. Impact of protein kinase PKR in cell biology: from antiviral to antiproliferative action. Microbiol Mol Biol Rev 70:1032–1060. doi:10.1128/MMBR.00027-06 17158706 PMC1698511

[29] Kawasaki H, Schiltz L, Chiu R, Itakura K, Taira K, Nakatani Y, Yokoyama KK. 2000. ATF-2 has intrinsic histone acetyltransferase activity which is modulated by phosphorylation. Nat New Biol 405:195–200. doi:10.1038/35012097 10821277

[30] Kim JH, Jang JY, Jang YJ. 2021. Human rhinovirus serotypes induces different immune responses. Virol J 18:232. doi:10.1186/s12985-021-01701-1 34838080 PMC8626727

[31] Winther B, Arruda E, Witek TJ, Marlin SD, Tsianco MM, Innes DJ, Hayden FG. 2002. Expression of ICAM-1 in nasal epithelium and levels of soluble ICAM-1 in nasal lavage fluid during human experimental rhinovirus infection. Arch Otolaryngol Head Neck Surg 128:131–136. doi:10.1001/archotol.128.2.131 11843719

